# Anoctamin-4 is a bona fide Ca^2+^-dependent non-selective cation channel

**DOI:** 10.1038/s41598-018-37287-y

**Published:** 2019-02-19

**Authors:** Nadine Reichhart, Simon Schöberl, Susanne Keckeis, Ahmad S. Alfaar, Christophe Roubeix, Magdalena Cordes, Sergio Crespo-Garcia, Akvile Haeckel, Norbert Kociok, Renate Föckler, Gabriele Fels, Anja Mataruga, Robert Rauh, Vladimir M. Milenkovic, Kerstin Zühlke, Enno Klussmann, Eyk Schellenberger, Olaf Strauß

**Affiliations:** 1 Experimental Ophthalmology, Department of Ophthalmology, Charité – Universitätsmedizin Berlin, a corporate member of Freie Universität, Humboldt-University, the Berlin Institute of Health, Berlin, Germany; 2 Department of Gastroenterology, Endocrinology, Rheumatology and Infectious Diseases, University Hospital Regensburg, Regensburg, Germany; 3 Department of Ophthalmology, Maisonneuve-Rosemont Hospital Research Centre, University of Montreal, Quebec, Canada; 4 Institute for Radiology and Children’s Radiology, Charité – Universitätsmedizin Berlin, a corporate member of Freie Universität, Humboldt-University, the Berlin Institute of Health, Berlin, Germany; 5 Experimental Ophthalmology, Eye Hospital, University Medical Center Regensburg, Regensburg, Germany; 6 Institute of Complex Systems, Zelluläre Biophysik (ICS-4), Forschungszentrum Jülich, Jülich, Germany; 7 Institut für Zelluläre und Molekulare Physiologie, Friedrich-Alexander-Universität Erlangen-Nürnberg (FAU), Erlangen, Germany; 8 Department of Psychiatry and Psychotherapy, Molecular Neuroscience, University of Regensburg, Regensburg, Germany; 9 Max Delbrück Center for Molecular Medicine Berlin (MDC), Berlin, Germany

## Abstract

Changes in cell function occur by specific patterns of intracellular Ca^2+^, activating Ca^2+^-sensitive proteins. The anoctamin (TMEM16) protein family has Ca^2+^-dependent ion channel activity, which provides transmembrane ion transport, and/or Ca^2+^-dependent phosphatidyl-scramblase activity. Using amino acid sequence analysis combined with measurements of ion channel function, we clarified the so far unknown Ano4 function as Ca^2+^-dependent, non-selective monovalent cation channel; heterologous Ano4 expression in HEK293 cells elicits Ca^2+^ activated conductance with weak selectivity of K^+^ > Na^+^ > Li^+^. Endogenously expressed Ca^2+^-dependent cation channels in the retinal pigment epithelium were identified as Ano4 by KO mouse-derived primary RPE cells and siRNA against Ano4. Exchanging a negatively charged amino acid in the putative pore region (AA702–855) into a positive one (E775K) turns Ano4-elicited currents into Cl^−^ currents evidencing its importance for ion selectivity. The molecular identification of Ano4 as a Ca^2+^-activated cation channel advances the understanding of its role in Ca^2+^ signaling.

## Introduction

The anoctamin (TMEM16) family includes ten homologs with two major functions: Ca^2+^-dependent ion channels and/or Ca^2+^-dependent scramblases^1–6^. The function as Ca^2+^-dependent Cl^–^ channels is well established for Ano1 and Ano2^6–12^. Controversial data suggest a function as a Ca^2+^-dependent cation channel and/or Cl^−^ channel function for Ano6^2,3,13–16^. Because of their scramblase activity, anoctamins (Ano3, Ano4, Ano6, Ano7, Ano9)^17^ may be able to regulate the activity of other endogenously expressed ion channels^2,5,16^. The scramblase activity of anoctamins may be responsible for the divergent observations not only in Ano6 but also in other anoctamins^5^. Scramblase activity at resting Ca^2+^ levels was observed for Ano4^17^; its ion channel function, however, has not been defined so far, although Schreiber and colleagues reported increased membrane currents after applying ionomycin to Ano4-transfected HEK293 cells^18^. Here, we determined that Ano4 is a bona-fide Ca^2+^-dependent cation channel when heterologously expressed in HEK293 cells or when endogenously expressed in retinal pigment epithelial cells.

## Results and Discussion

**Figure 1:**
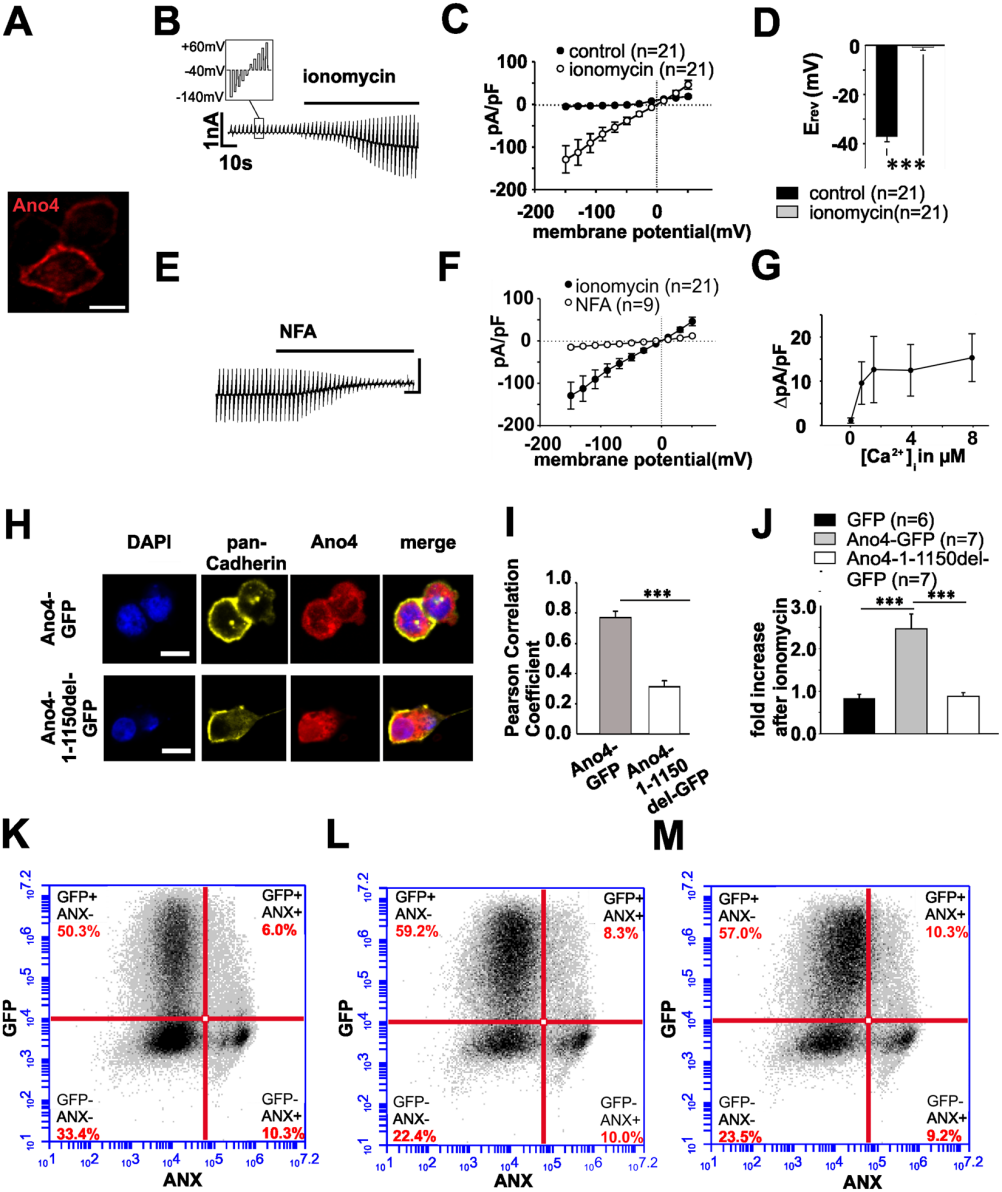
Heterologous expression of Ano4 elicits Ca^2+^-dependent cation conductance that is sensitive to fenamates. (**A**) Confocal image of HEK293 cells expressing Ano4-c-Myc. Staining with an antibody against c-Myc (red). Scale bar represents 10 µm. (**B**) Raw currents through HEK293 cells heterologously expressing Ano4 before and after the application of ionomycin (1 µM) as indicated by the bar. Small box illustrating the stimulation protocol applied to the cells: 10 voltage steps between −140 mV and +60 mV of a 50 ms duration applied every 2.5 s. (**C**) Current density-voltage plot of HEK293 cells expressing Ano4 before (filled circles) and after the application of ionomycin (open circles). (**D**) Reversal potential of the cells in C before (black bar) and after the application of ionomycin (grey bar). (**E**) Raw currents through HEK293 cells expressing Ano4 in an ionomycin-containing Ringer solution before and after the application of the fenamate niflumic acid (NFA, 100 µm). Application is indicated by the bar. (**F**) Current density-voltage plot before (filled circles) and after the application of NFA (open circles). (**G**) Spline curve of change of current density (ΔpA/pF) of Ano4 transfected HEK293 cells at different [Ca^2+^]_i_. (**H**) Confocal images of HEK293 cells transfected with Ano4-GFP (upper panel) or Ano4-1-1150del-GFP (lower panel). Staining against pan-Cadherin (yellow) and Ano4 (red). Nuclei were stained with DAPI. Scale bar represents 10 µm. (**I**) Bar chart representing the Pearson Correlation Coefficient between pan-Cadherin and Ano4 pixels (transfection and immunostaining according to (**H**)). (**J**) Ionomycin-mediated increase in current density in cells transfected with GFP (black bar), Ano4-GFP (grey) and Ano4-1-1150del-GFP (white bar). (**K–M**) Assessment of scramblase activity by FACS sorting of annexin A5-labeled HEK293 cells transfected with GFP alone (**K**), with Ano4 plus GFP under control conditions (**L**) or after the application of ionomycin (1 µM). (**M**) X-axis: Fluorescence intensity of Anx A5-6S-IDCC (log); Y-axis: Fluorescence intensity of GFP (log). The right upper square represents the transfected, Annexin A5-positive cell fraction. Values are given as mean ± SEM. ^***^p < 0.001.

We used HEK293 cells as an expression system for Ano4 in which Ano6 fails to induce phosphatidyl-serine scrambling^14^. Heterologously expressed full length Ano4 localizes to the plasma membrane as determined by immunohistochemistry (Fig. 1A,H). HEK293 cells over-expressing Ano4 showed increased membrane conductance when intracellular free Ca^2+^ was increased by the extracellular application of ionomycin (Fig. 1B,C). Under these experimental conditions, the reversal potential of the current changed from −37 mV to −0.9 mV, which is a change from the predicted equilibrium potential for Cl^−^ to that for monovalent cations (Fig. 1D). The slow response to ionomycin seems to be a characteristic feature of anoctamins. In a very similar approach to ours, Stöhr *et al*.^8^ demonstrated an approx. 25 s latency in current density increase upon ionomycin stimulation of Ano2 transfected HEK cells. In another publication, Yang *et al*.^9^ also described 30 s latency of Ano 1 current increase upon increase of intracellular Ca^2+^. GFP control-transfected cells showed mild outwardly rectifying currents that were not increased by ionomycin application (Suppl. Fig. 1A–C,G). Untransfected HEK293 cells did not show Ano4 expression. There was no current density increase upon stimulation of non-transfected cells with ATP or GFP transfected cells with ionomycin (Suppl. Fig. 1K,L)

**Figure 2:**
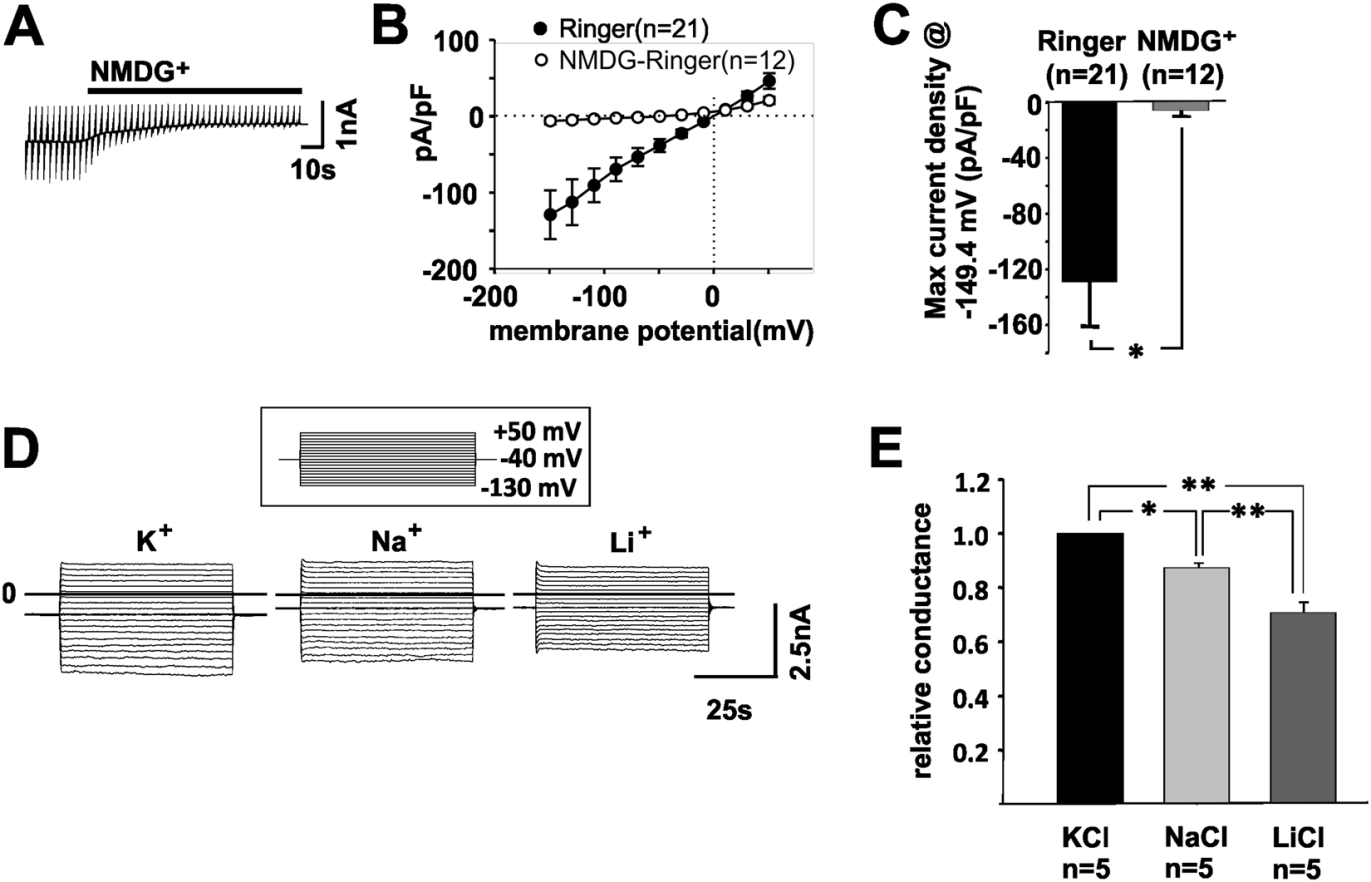
Analysis of the cation conductance of heterologously expressed Ano4. (**A**) Raw currents through HEK293 cells heterologously expressing Ano4 before and after the substitution of extracellular cations by NMDG^+^. Application is indicated by the bar. (**B**) Current density-voltage plot before (filled circles) and after the application of NMDG (open circles). (**C**) Maximum current density of the cells in B at +60 mV before (black bar) and after the application of NMDG (grey bar). ^*^p < 0.05. (**D**) Raw current of HEK293 cells expressing Ano4 stimulated by ionomycin; the pipette solution and bath solution contained equimolar concentrations of K^+^ (left), then after exchange by equimolar Na^+^ (middle), and after exchange by an equimolar concentration of Li^+^ (right). Currents were elicited by electrical stimulation shown in the insert (from a holding potential of −40 mV, the cell was first depolarized by 9 voltage steps of 10 mV, followed by nine voltage steps of −10 mV). (**E**) Relative conductance to K^+^ (black bar): Na^+^ (light grey bar) and Li^+^ (dark grey bar) calculated from experiments shown in 2 A. Values are given as mean ± SEM.^*^p < 0.05; ^**^p < 0.01.

The Ano4-dependent current was blocked by the application of the fenamate niflumic acid, which is known as a potential blocker of Ano1^9–11^ (Fig. 1E,F). Using pipette solutions containing different Ca^2+^ concentrations ([Ca^2+^]_i_), a [Ca^2+^]_i_-response curve was measured resulting in a [Ca^2+^]_i_ for half-maximal activation of approx. 700 nM (Fig. 1G). Ionomycin application did not significantly increase the surface expression of Ano4 in HEK 293 or ARPE-19 cells. (Suppl Fig. 7), an alternative or concurrent mechanism that might have explained the current density increase after ionomycin application. Removal of the first transmembrane domain of Ano4 (Ano4-1-1150del-GFP) led to a significant reduction of both membrane localization (as demonstrated by a reduction of the PCC between pan Cadherin and Ano4) and density of Ca^2+^-evoked currents (Fig. 1H–J). In addition, membrane localization of Ano4-GFP was further demonstrated via a biotinylation assay (Suppl. Fig. 4E). The rather small current density increases in cells transfected with GFP-tagged full length Ano4 (less than 3 fold) compared to transfection with untagged Ano4 (more than 25 fold, Fig. 1C) might be explained by the GFP tag hampering or interfering with channel activity. Ano4 expression in HEK293 cells did not substantially change the scramblase activity under resting or under elevated [Ca^2+^] as examined by phosphatidyl-serine (PS) externalization using annexin A5, which binds with high affinity to PS (Fig. 1K–M, Supp. Fig. 2). This applies for both the time needed for current activation (5 min) and a longer period (20 min). Experiments in which the non-permeable NMDG^+^ replaced extracellular monovalent cations after full activation of Ca^2+^-dependent conductance further proved the cation channel activity (Fig. 2A–C). Under these conditions, the inward currents disappeared completely, whereas the outward currents decreased mildly. The mild reduction in the outward current could be explained by the observation that extracellular cations influence the gating of anoctamins^18^. Control experiments with the Cl^−^ channel Ano2-expressing HEK293 cells showed no changes in the Ca^2+^-dependent conductance (Suppl. Fig. 1D–I). The ion selectivity of Ano4 was further analyzed in the presence of equimolar extracellular concentrations of KCl, NaCl or LiCl. The calculation of the relative conductance for K^+^ (Fig. 2D,E) revealed weak selectivity, with a conductance sequence of K^+^ > Na^+^ > Li^+^, which is consistent with Eisenman I–VI permeability sequences. Thus, several lines of evidence demonstrate that Ano4 elicited Ca^2+^-dependent cation conductance.

**Figure 3:**
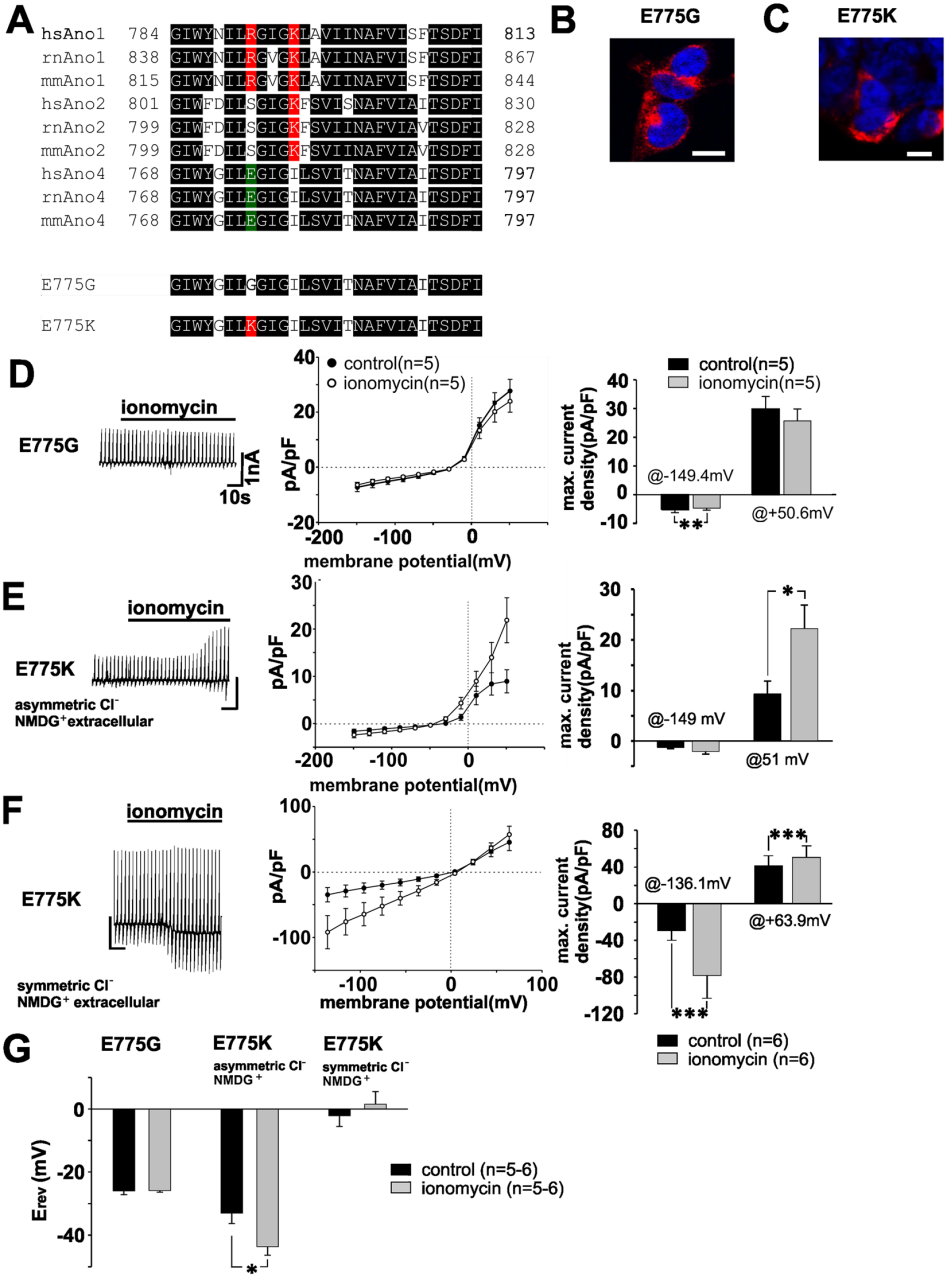
E775 is a crucial amino -acid for Ano4 ion selectivity. (**A**) *Upper part:* Sequence alignment of amino acids (between aa 768 and 797 in murine Ano4) of mouse, rat and human Ano1, 2 and 4. Conserved amino acids are colored in black. Negatively charged amino acids in Ano4 are highlighted in green, and positively charged amino acids in Ano1 and 2 are highlighted in red. *Lower part:* Corresponding sequence of the two mutations E775G and E775K. hs: *Homo sapiens*; mm: *Mus musculus*; rn: *Rattus norvegicus*. Detailed sequence information is shown in Supp. Fig. 3 and Supp. Table 1. (**B**) Confocal image of HEK293 cells expressing E775G-c-Myc. Anti-Ano4 antibody staining (red). Nuclei stained with DAPI. Scale bar represents 10 µm. (**C**) Confocal image of HEK293 cells expressing E775K-c-Myc. Staining with an antibody against Ano4 (red). Nuclei stained with DAPI. Scale bar represents 10 µm. (**D**) Left panel: Raw currents through HEK293 cells heterologously expressing E775G in Ringer solution before and after the application of ionomycin (1 µM) as indicated by the bar. Middle panel: Current density-voltage plot before (filled circles) and after the application of ionomycin (open circles). The values are given as the mean ± SEM. Right panel: Maximum current density at −140 mV and at +60 mV before (black bars) and after the application of ionomycin (grey bars). ^**^p < 0.01. (**E**) Left panel: Raw currents through HEK293 cells heterologously expressing E775K (extracellular Ringer-NMDG and intracellular asymmetric Cl^−^) before and after the application of ionomycin (1 µM) as indicated by the bar. Middle panel: Current density-voltage plot before (filled circles) and after the application of ionomycin (open circles). The values are given as the mean ± SEM. Right panel: Maximum current density at −140 mV and at + 60 mV before (black bars) and after the application of ionomycin (grey bars). ^*^p < 0.05. (**F**) Left panel: Raw currents through HEK293 cells heterologously expressing E775K (extracellular Ringer-NMDG and intracellular symmetric Cl^−^) before and after the application of ionomycin (1 µm) as indicated by the bar. Middle panel: Current density-voltage plot before (filled circles) and after the application of ionomycin (open circles). The values are given as the mean ± SEM. Right panel: Bar chart illustrating the maximum current density at −140 mV and at + 60 mV before (black bars) and after the application of ionomycin (grey bars). ^***^p < 0.001. (**G**) Reversal potentials of the data shown in E (left), F (middle) and G (right) before (black bars) and after (grey bars) the application of ionomycin. ^**^p < 0.01.

To substantiate that the cation conductance originates from the Ano4 protein, we used a site-directed mutagenesis approach. To identify a target, we searched in vertebrate sequences for conserved positively charged amino acids in Ano1 and Ano2 that are conserved negatively charged amino acids in Ano4. In Ano1 and Ano2, we found two sites with conserved positively charged amino acids that were also found by Yang *et al*.^9^ in Ano1 and by Yang *et al*.^14^ in Ano6. In one site, Ano1, Ano2 and Ano4 all contain several positively charged conserved amino acids. Thus, this region was not of interest for further studies. The other site represents a stretch of 8 amino acids containing predominantly positively charged amino acids with a conserved K (Fig. 3A and Suppl. Fig. 3; Suppl. Table 1). In the same area, Ano4 contains only a conserved negatively charged amino acid E and no positively charged amino acids. Furthermore, in the mouse Ano4, this E localizes at position 775, close to a stretch of amino acids related to the pore function of Ano1 that is based on generated mutants with conductivity changes^5,6,12,19–21^, and according to our own sequence analysis. We investigated the functional role of 775-E in Ano4 by generating two different variants using site-directed mutagenesis. In one variant, 775-E was exchanged with a non-charged amino acid (E775G), and in the other variant, 775-E was exchanged with a positively charged amino acid (E775K). When expressed in HEK293 cells, both variants showed plasma membrane localization comparable to wild-type Ano4 (Fig. 3B,C, Suppl. Fig. 4A–C). E775G-expressing HEK293 cells showed no Ca^2+^-activated currents (Fig. 3D). E775K-expressing HEK293 cells showed activation of outwardly rectifying currents when the intracellular Cl^−^ concentration was lower than the extracellular Cl^−^ concentration (Fig. 3E). The reversal potential of the Ca^2+^-activated current was at −43.6 mV, which is close to the equilibrium potential for Cl^−^ under these conditions (Fig. 3G). When using a pipette solution with equimolar Cl^−^ intra- and extracellular concentrations, in addition to the replacement of extracellular monovalent cations by NMDG^+^, a current with a linear current/voltage relationship and a reversal potential of 1.5 mV was activated in Ano4-E775K expressing cells (Fig. 3F,G). The currents measured in Ano4-E775K-expressing cells were completely insensitive to extracellular NMDG^+^ replacement for cations and followed exactly the reversal potential for Cl^−^. Thus, we turned the Ano4 wild-type cation channel into a pure Cl^−^ channel by E775K amino acid exchange, which demonstrates the essential role of E775 in Ano4 ion selectivity. In summary, heterologously expressed Ano4 functions as a bona-fide Ca^2+^-dependent cation channel.

**Figure 4:**
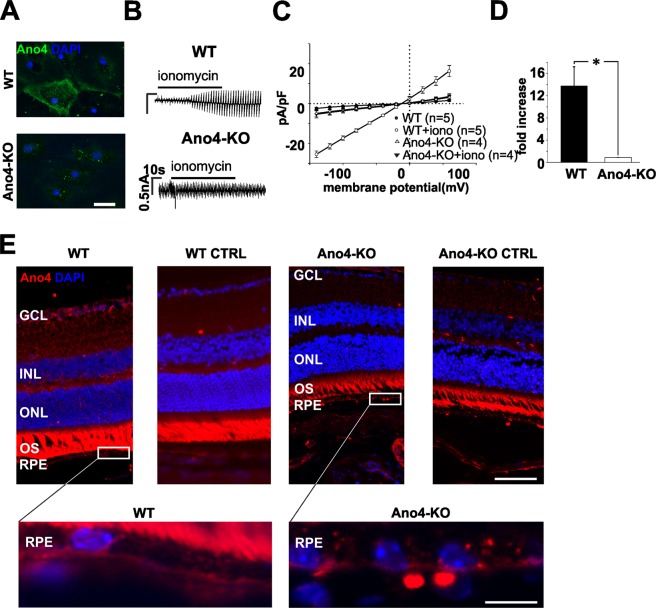
KO of Anoctamin 4 leads to an abolishment of Ca^2+^-dependent cation conductance in mouse primary RPE cells. (**A**) Confocal image of wildtype (upper panel) or Ano4-KO (lower panel) primary RPE cells stained with an antibody against Ano4. Scale bar represents 25 µm. (**B**) Raw currents through primary RPE cells of wildtype (upper panel) or Ano4-KO (lower panel) before and after the application of ionomycin (1 µM). Application is indicated by the bar. (**C**) Current density-voltage plot before and after the application of ionomycin in WT (black) and Ano4-KO (white) cells. (**D**) Fold increase of total current density after the application of ionomycin in WT cells (black bar) or Ano4-KO (white bar) cells). (**E**) Staining of a murine sagittal section of wildtype (1^st^ column) or Ano4-KO (3^rd^ column) with an antibody against Ano4. 2^nd^ and 4^th^ column show the respective isotype controls (incubation only with the secondary antibody). Nuclei were stained with DAPI. Upper panel: Scale bar represents 50 µm. GCL: ganglion cell layer; ONL: outer nuclear layer; INL: inner nuclear layer; IS: inner segments, OS: outer segments; RPE: retinal pigment epithelium. Lower panel: Magnification of the upper panel in the RPE. Scale bar represents 10 µm. The values are given as mean ± SEM. ^*^p < 0.05.

To demonstrate the relevance of endogenously expressed Ano4 for Ca^2+^ dependent conductance, we used primary retinal pigment epithelium (RPE) cells, for which a fenamate-sensitive and Ca^2+^-dependent cation conductance, activated in response to stimulation by ATP, has been reported (18). RPE cells of wildtype (WT) mice showed Ano4 expression predominantly in the cell membrane, whereas RPE cells of Ano4-KO mice were lacking Ano4 (Fig. 4A). RPE cells derived from WT mice revealed an almost 14-fold increase of total current density in response to ionomycin, whereas KO of Ano4 led to an abolishment of the response to ionomycin (Fig. 4B–D). Immunohistochemical staining of mouse retinal sagittal sections revealed Ano4 expression at the basolateral side of the RPE. In the retina, parts of the inner nuclear, outer plexiform and ganglion cell layer were positive for Ano4. Considering the negative control, the staining of the outer segments of the photoreceptors is unspecific (Fig. 4E). Immunocytochemistry in heterologous expression further proved the specific detection of Ano4 by our antibody (Supp. Fig. 5A).

**Figure 5:**
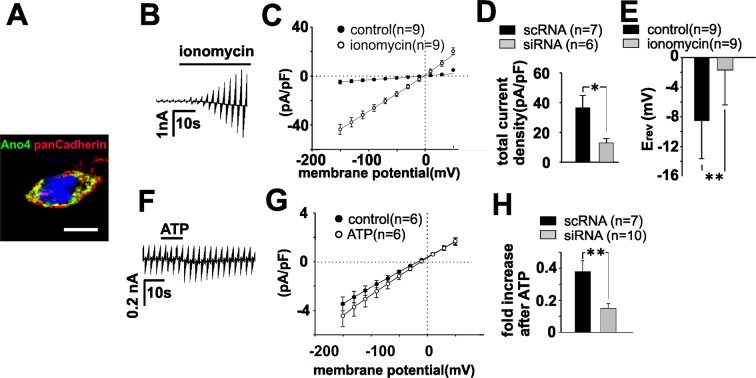
Endogenously expressed Ano4 is responsible for Ca^2+^-dependent cation conductance in RPE cells. (**A**) Confocal image of an ARPE-19 cell expressing Ano4. Staining with an antibody against Ano4 (green). Cell membrane was stained with ZO-1 (red). Scale bar represents 10 µm. (**B**) Raw currents through ARPE-19 cells before and after the application of ionomycin (1 µM). Application is indicated by the bar. (**C**) Current density-voltage plot before (filled circles) and after the application of ionomycin (open circles). (**D**) Comparison of the total current density in cells transfected with scRNA (black bar) and siRNA (grey bar). (**E**) Reversal potentials of the data shown in C before (black bars) and after (grey bars) the application of ionomycin. (**F**) Raw currents through ARPE-19 cells before and after the application of ATP (500 µM). (**G**) Current density-voltage plot before (filled circles) and after the application of ATP (open circles). (**H**) ATP-mediated increase in current density in cells transfected with scRNA (black bar) and siRNA (grey bar). The values are given as mean ± SEM. ^*^p < 0.05; ^**^p < 0.01.

To find more evidence of the function of Ano4 as Ca^2+^-dependent cation channel, we investigated ARPE-19 cells, that endogenously express Ano4 (Fig. 5A), by means of a siRNA approach. Membrane currents were recorded in ARPE-19 cells in the whole-cell configuration, with a higher Cl^−^ concentration in the bath than that in the pipette solution. Ionomycin application led to the activation of a membrane conductance with a reversal potential of −1.7 mV, indicating the activation of cation conductance (Fig. 5B–E). Comparable currents were transiently activated in response to application of 500 µM ATP (Fig. 5F–H). At the peak of their activation, these currents showed almost linear current/voltage relationships with a reversal potential of 0 mV. When using siRNA knockdown, Ano4 expression was reduced by 50% in ARPE-19 cells compared to that in cells treated with non-targeted siRNA (Suppl. Fig. 6). In ARPE-19 cells treated with Ano4-targeted siRNA, the ionomycin-activated current density was reduced by 64% (Fig. 5D) and the ATP-stimulated current density increase was reduced by 60% (Fig. 5H). The responses to ATP as shown in Fig. 5F–H are much smaller than those to ionomycin. This can be explained by the fact that changes in intracellular Ca^2+^ elicited by ionomycin are much larger than changes induced by ATP. In RPE cells, ATP acts via P2Y receptors that increase intracellular free Ca^2+^ solely by release of Ca^2+^ from cytosolic stores that can release only a limited amount of Ca^2+^ to the cytoplasm and lasts only a couple of seconds. In contrast to that, ionomycin forms a pore into the plasma membrane and extracellular Ca^2+^ becomes the source of Ca^2+^ to increase cytosolic free Ca^2+^. In summary, siRNA knockdown of Ano4 in ARPE19 cells demonstrated that endogenously expressed Ano4 protein, provides a Ca^2+^-activated non-selective monovalent cation current.

Thus, the anoctamin family includes both anion and cation channels. Since the first x-ray crystallographic analysis, a more detailed structure/function correlation of different anoctamins is available. Site-directed mutagenesis identified the localization of amino acids that are required for Ca^2+^-binding or that interfere with conductance properties in several anoctamins. Work from different groups demonstrated that a stretch of amino acids between the positions 559 and 698, depending on the anoctamin family member, is associated with rectification properties^12,14,22^. Yang *et al*.^14^ identified K584 to be of importance for permeation selectivity, a finding that could, however, not be confirmed by Jeng *et al*.^22^; Lim *et al*.^12^ showed a loss of pore function by mutating the K588 in Ano1. Modeling of the three-dimensional structure of anoctamins based on x-ray crystallography predicts that the helices 3–8 form a region that includes both Ca^2+^-binding sites and the pore^4–6^. Amino acid positions targeted by site-directed mutagenesis approaches with effects on conductance properties confirm this. Our comparative search for conserved positively or negatively charged amino acids in the sequences in Ano1 and Ano2 versus Ano4 resulted in the identification of the positions around R765/K769 (Ano1) and E775 (Ano4). Since mutating amino acids around K769 did not result in reproducible effects on ion selectivity, we focused on E775 that appeared to determine cation conductance. The mutation we inserted in mAno4 corresponds to amino acid position 765 in mAno1. In both Ano1 and Ano4, these amino acid positions localize to the 9^th^ helix at its cytosolic end, close to the pore-forming helices composed of helix 3–8 in the three-dimensional structure^6^. These amino acids positions might define reliable targets for analysis of the ion channel functions of other members of the anoctamin family.

## Materials and Methods

### Mouse RPE cell isolation

Experiments were designed and performed in accordance to the ARVO Statement for the Use of Animals in Ophthalmic and Vision Research and approved by the local authorities (Landesamt für Gesundheit und Soziales LaGeSo, T0009/17). Primary mouse RPE cells of 11–14 months old Ano4KOCfa mice (Ano4-KO mice, genetic background: C57/Bl6) and age-matched C57/Bl6 controls were isolated as described previously^23^. KO-mutation of Ano4 was introduced via TALEN mediated excision of bases by Cyagen Biosciences (B6.Slc1A7tm1Cfa Santa Clara, CA, USA). After enucleation of the eyes and incubation in DMEM (supplemented with 0.1% gentamycin (Merck Millipore, Darmstadt, Germany) and 4.5 g/L of glucose) overnight at RT, the eyes were incubated in 1.5 mg/ml of trypsin (Thermo Fisher Scientific, Berlin Germany) and 2 mg/ml of collagenase (Worthington, Columbus, USA) for 60 min at 37 °C. After detachment of the RPE sheets from the retina, trypsin was applied to dissociate RPE sheets and to obtain single cells. Cells were placed on glass coverslips and maintained at 37 °C, with relative humidity of 95% and 5% CO_2_. 7–10 days after seeding, Patch-Clamp experiments were performed.

### Cell culture and transfection

The human cell lines HEK-293 (CRL-1573; ATCC, Wesel, Germany) and ARPE-19 (CRL-2302; ATCC) were cultured in 100-mm culture dishes in Dulbecco’s Modified Eagle’s Medium (D-MEM)/Nutrient F-12 Ham (Sigma; Schnelldorf; Germany) supplemented with 10% (v/v) fetal calf serum (Biochrom, Berlin, Germany) and 1% (v/v) penicillin-streptomycin (Biochrom) at 37 °C, with relative humidity of 95% and 5% CO_2_ concentration. HEK-293 cells seeded on 15-mm glass cover slips were co- transfected with Ano2 or Ano4 or D and YFP or GFP expression constructs. All transfections were carried out using Lipofectamine Transfection Reagent (Invitrogen, Darmstadt, Germany) following the manufacturer’s protocol.

### Cloning of Ano4 and Ano2

The mammalian expression construct of mouse Ano2 in pcDNA3 (Invitrogen, Karlsruhe, Germany) has been previously described^8^. The full-length coding sequence of mouse Ano4 was RT-PCR amplified from mouse retinal RNA with oligonucleotide primer pair Ano4F5-KpnI (5′-ggtaccCAATAAAAATGGAGGCCAGCT-3′) and Ano4R4-HindIII (5′-aagcttTGGCCACTCATTGTGATGTG-3′). The 2885-bp PCR product was cloned into the KpnI and HindIII sites of pCEP1.4 vector in-frame with a C-terminal rhodopsin (Rho)-1D4 tag^24^ and sequenced. To obtain comparable expression rates of Ano2, Ano4 cDNA was cloned into the KpnI and AgeI sites of pcDNA3 (in-frame with a C-terminal Myc tag) or into pEGFPN1 (Takara, Saint-Germain-en-Laye, France). Deletion of the first transmembrane domain of Ano4 (bp 1–1150) in pEGFPN1 was introduced by the following forwards: 5′-CAGCCCATTGACCTGGT-3′ and reverse primer: 5′-GATGTCGCTGTCCTGCG-3′(Ano4-1-1150del-GFP).

### Site directed mutagenesis of Ano4

Sequence alignments of ANO 1, 2 and 4 (for sequences see Supp. Fig. 3) were performed using ClustalW (European Bioinformatics Institute, Cambridge, United Kingdom). Mutations were generated on the full-length mouse Ano4 (as described above): (NM_001277188.1; NP_001264117.1; 955 amino acids), applying the QuikChange II site-directed mutagenesis kit (Stratagene, Santa Clara, USA). For the exchange of glutamate (E) at AA position 775 to the positively charged lysine (K) the following primer pair was used: E775K_F:5′-GGAATTTGGTATGGAATTCTCaAAGGCATTGGGATTCTGTCTG-3′ and E775K_R: 5′-CAGACAGAATCCCAATGCCTTtGAGAATTCCATACCAAATTCC-3′, and for the exchange of glutamate to the neutral AA glycine (G): E775G_F: 5′-GGTATGGAATTCTCGGAGGCATTGGGATTCTG-3′. E775G_R: 5′-CAGAATCCCAATGCCTCCGAGAATTCCATACC-3′.

The final Ano4 constructs were fully sequenced to ensure the targeted mutagenesis had occurred correctly and to exclude the presence of undesired sequence alterations.

### Patch clamp recordings

Whole-cell recordings were performed at room temperature on single YFP positive cells. Patch pipettes with a pipette resistance of 3–5 MΩ were pulled from borosilicate glass tubes using a DMZ Universal Puller (Zeitz, Augsburg, Germany). Whole-cell currents were measured using an EPC 7 and EPC 10 patch-clamp amplifier and TIDA software (HEKA Electronics, Ludwigshafen, Germany). Currents were filtered at 2.9 kHz with a low-pass Bessel filter. Access resistance was compensated to values lower than 10 MΩ.

To examine basic conductance ionomycin-free and ionomycin-containing (1 µM) extracellular Ringer solution was used (Fig. 1) (mM) (145NaCl, 0.4 KH_2_PO_4_, 1.6 K_2_HPO_4_, 1.3 Ca-gluconate, 1 MgCl_2_, 5 glucose, pH 7.2). Pipette solution contained 95 mM K-gluconate, 30 mM KCl, 4.8 mM Na_2_HPO_4_, 1.2 mM NaH_2_PO_4_, 0.3 mM Ca-gluconate, 1 mM EGTA, 5 mM glucose, 3 mM (Na)_2_ATP, pH 7.2. The final concentration of free Ca^2+^ in the pipette solution was 100 nM. To determine the calcium dependency of the current, intracellular [Ca^2+^] was increased to 800 nM, 1.6 µM, 4 µM and 8 µM. The voltages measured in experiments using Ringer solution were corrected for liquid junction potential (9.4 mV). In experiments for examination of inward currents, Ringer solution was replaced by NMDG^+^-Ringer solution (145 mM NMDG-Cl, 0.4 mM KH_2_PO_4_, 1.6 mM K_2_HPO_4_, 1.3 mM Ca-gluconate, 1 mM MgCl_2_, and 5 mM glucose; pH 7.2). In order to study membrane conductance in the absence of K^+^ and Na^+^, intracellular K^+^ and Na^+^ were almost replaced by NMDG^+^ (95 mM NMDG-CH_3_SO_3_, 30 mM KCl, 15 mM HEPES, 0.81 mM Ca-gluconate, 1 mM EDTA, 5 mM glucose, and 3 mM ATP, pH 7.2). Potentials were corrected for liquid junction potential of 2.5 mV. To analyze the cation conductance ratios, extracellular solutions contained 125 mM KCl, 125 mM NaCl or 125 mM LiCl respectively, in addition to 25 mM mannose, 5 mM glucose, 1 mM MgCl_2_, 1.3 mM Ca-gluconate, 10 mM Hepes (pH = 7.2). For calculation of relative conductance, the intracellular solution contained an equal amount of K^+^ with KCl 125 mM, 1 mM EDTA, 0.81 mM Ca-gluconate, 15 mM Hepes, 5 mM glucose and 3 mM ATP (pH = 7.2). To achieve symmetric Cl^−^ concentrations in the pipette and the bath solution K-Gluconate was substituted by KCl (resulting in an intracellular Cl^−^ concentration of 125 mM). The membrane capacitance was 21 ± 5.5 (n = 10) pF for YFP-transfected cells, 17.3 ± 1.3 (n = 49) pF for Ano4-transfected cells and 9.7 ± 0.6 (n = 18) pF for TMEM16B transfected cells. For recordings of Ano4-GFP and Ano4-1-1150del-GFP the bath solution contained (in mM): NaCl 145, KH_2_PO_4_ 0.4, K_2_HPO_4_ 1.6, Ca-Gluconate 1.3, MgCl_2_ 1, glucose 5. The pipette solution contained (in mM): K-gluconate 88, KCl 30, Na_2_HPO_4_ 4.8, NaH_2_PO_4_ 1.2, Ca gluconate 0.3, EGTA 1, Na_2_ATP 3. For siRNA experiment, ARPE-19 cells were superfused with an extracellular solution containing 136.43 mM NaCl, 1.1 mM Na_2_HPO_4_, 4.17 mM NaHCO_3_, 0.89 mM MgCl_2_, 0,95 mM CaCl_2_, 5.8 mM TEACl, 25 mM HEPES, and 11.1 mM glucose; pH 7.2. ATP was applied to the extracellular solution in a concentration of 500 µM. Pipette solution contained 80 mM Cs-MeSO_3_, 30 mM NaCl, 2 mM MgSO_4_, 0.5 mM CaCl_2_, 5.5 mM EGTA, and 10 mM HEPES; pH 7.2. Potentials were corrected for liquid junction potential of 10.7 mV.

For recordings of primary mouse RPE cells, the following solutions were used according to Wollmann *et al*.^25^: bath solution (in mM): NaCl 130, TEACl 3, CaCl_2_ 1.5, MgCl_2_, NaHCO_3_ 14, NaHPO_3_ 1, HEPES 33, Glucose 5.5. Pipette solution (in mM): CsMeSO_4_ 80, NaCl 30, MgSO_4_ 2, CaCl_2_ 0.5, EGTA 5.5, HEPES 10.

### Measurement of scramblase activity

Scramblase activity was measured by binding of fluorescent-labeled annexin A5 to exposed phosphatidyl-serine. HEK cell pellets (0.5 to 1 × 10^6^ cells) were washed once with 500 µl PBS and twice with 500 µl binding buffer (1.3 mM CaCl_2_, 10 mM HEPES, 150 mM NaCl, 5 mM KCl, 1 mM MgCl_2_, pH 7.4). Cys-annexin-A5 (NeXins Research, The Netherlands) was labeled with maleimide-6S-IDCC (Mivenion GmbH, Berlin, Germany) at the singular cysteins as described elsewhere^26^. 0.25 µg of cys-anxA5-6S-IDCC was added to each sample and incubated in the dark for 10 min. Flow cytometry measurements of annexin A5 binding and GFP-expression were performed using a BD Accuri C6 flow cytometer (BD Biosciences, Heidelberg, Germany) according to the manufacturer’s instructions.

### siRNA knockdown of endogenous Ano4 in ARPE-19 cells

For specific knockdown of human ANO4 in ARPE-19 cells, a mixture of 3 commercially available AF488-labelled stealth siRNAs (HSS133556, HSS133557, HSS174644, Life Technologies, Darmstadt, Germany) was used. A scrambled sequence siRNA double-stranded oligomer not homologous to any known gene, also labelled with AF488 (Invitrogen) served as control. Transfections were carried out with Lipofectamine 2000 (Invitrogen) according to the manufacturer’s instructions. Cells were subjected to RNA isolation or patch clamp 24–36 h after transfection.

#### RNA-Isolation and qPCR

Total RNA from ARPE-19 cells was prepared from 3 subconfluent wells of a 6 well plate. Cells were harvested by accutase (Sigma) and RNA was subsequently isolated using RNeasy Mini Kit (Quiagen, Hilden, Germany) according to the manufacturer’s instructions. RNA was reverse transcribed using Quantitect Reverse Transcription Kit (Quiagen) according to the manufacturer’s protocol. The mRNA levels of ANO4 (hsAno4_F: 5′-TTGTAAGGCGGTACTTTGGAGA-3′ and hsAno4_R: 5′-ATCCAGAGTGGTGACGCCATA-3′) and the housekeeping gene GAPDH (hsGAPDH_F: 5′-TCAACGACCACTTTGTCAAGCTCA-3′; hsGAPDH_R: 5′-GCTGGTGGTCCAGGGGTCTTACT-3′)were compared in scRNA and siRNA transfected ARPE-19 cells by Real Time RT-PCR using SYBR Green PCR Master Mix (Quiagen) on an Rotor-Gene Q (Qiagen). Experiments were repeated four times. Data were analyzed with the Rotor-Gene Q software 2.1.0 (Qiagen). According to Willems *et al*.^27^ the ddCt values went through a series of sequential corrections, including log transformation, mean centering, and autoscaling, to draw statistically reliable conclusions.

### Cell surface biotinylation essay

Biotinylation was performed according to Alken *et al*.^28^. In short, HEK293, untransfected and transfected with Ano4-GFP or GFP respectively were incubated with 1 mg/ml Biotin (Sigma) in biotinylation buffer for 60 min at 4 °C. After several washes with ice cold buffer, cells were incubated in Quenching Buffer for 10 min at 4 °C while shaking. Cells were resuspended in a Lysisbuffer, supplemented by protease inhibition cocktail according to Rosenthal *et al*.^29^. After sonication, the lysate was incubated at 37 °C for 30 min and centrifuged at 13000 rpm at 4 °C for 10 min. 2 mg of protein were incubated with 300 µl of Streptavidin Agarose (Fisher Scientific) ON at 4 °C while rotating. After several washing steps, loading buffer was added to the Agarose fraction and samples were incubated at 70 °C for 10 min. Samples were objected to a 10% SDS gel and Western blot was performed using a Turbo Blot unit (Biorad). After blocking the membrane in 5% milk in TBS Tween, a primary anti GFP antibody was applied overnight at 4 °C. After incubation with species appropriate HRP conjugated secondary antibody proteins were visualized with a Lumi-Light substrate solution (Roche Applied Science, Penzberg, Germany) using the Lumi Imager F1 (Roche Applied Science).

#### Protein extraction and western blot

Transfected ARPE-19 were removed from culture dishes using accutase. ARPE-19 cells were subsequently homogenized using a special, ice-cold lysis Buffer and protease inhibitor according to Rosenthal *et al*.^29^. After 10 s ultrasound, lysates were incubated on ice for 20 min and centrifuged for 20 min at 13,000 × g at 4 °C. After adding appropriate amount of Lämmli buffer and a boiling step at 95 °C for 3 min while shaking, 30 µg of each sample was subjected to SDS-PAGE containing 8% polyacrylamide. After transfer of the proteins to PVDF membranes (Biorad, München, Deutschland), and incubation in 5% non-fat dry milk powder for 1 hour at room temperature to minimize unspecific binding of the antibody, the primary antibody (anti-Ano4, 1:500) was applied over night at 4 °C. β-Actin (1:5000, mouse monoclonal, Sigma, Taufkirchen, Germany) served as a loading control. Blots were visualized using HRP-conjugated secondary antibodies (1:5000 goat anti rabbit or mouse IgG (GE Healthcare, Buckinghamshire, UK) and an enhanced chemiluminescence kit (Biorad). The images were digitalized using Molecular Imager ChemiDoc XRS (Biorad). Densitometric analysis was performed using Quantity One 1-D Analysis Software (Biorad) to determine the extent of siRNA mediated reduction in Ano4 expression compared to scRNA transfection.

### Immunostaining of Ano4

*Immunocytochemistry:* Primary mouse RPE cells or transfected HEK and ARPE-19 cells on glass cover slips were fixed with 4% (w/v) para-formaldehyde for 10 min at room temperature. After three washing steps with 1 × TBS, cells were incubated in a blocking solution containing (5% (v/v) BSA for 45 min. Due to the c-Myc tag in the TMEM-plasmids ARPE-19 and HEK cells could be labeled overnight at 4 °C with primary antibody against c-Myc (mouse monoclonal, 1:500; Santa Cruz Biotechnology, Santa Cruz, USA) or against Ano4 (rabbit polyclonal, 1:100; Davids Biotechnology, Regensburg, Germany). For visualizing YFP fluorescence, anti GFP (mouse monoclonal, 1:250, Abcam, Cambridge, UK) was used. After incubation with fluorescence conjugated species appropriate secondary antibodies (goat anti-mouse AF 546 and donkey anti-rabbit AF488 1:10 000; Life Technologies) cells were mounted onto glass slides and examined using an Axio Imager 2 and Zen lite 2012 Software (Zeiss, Jena, Germany). Partial merge of staining against Ano4 and c-Myc in HEK293 transfected with Ano4-c-Myc illustrates the ability of the custom-made antibody to detect Ano4. C-Myc staining is more abundant in the cell membrane and the cytosol since HEK293 endogenously express c-Myc (Supplement Fig. 5A). *pan*-Cadherin (mouse monoclonal, 1:250, abcam) was used to stain the cell membrane. Pearson Correlation Coefficient was determined using Image J software to detect co-localization of Ano4 and pan-Cadherin in the cell membrane (Rasband, W.S., ImageJ, U.S. National Institutes of Health, Bethesda, Maryland, USA, 1997–2015).

#### Immunohistochemistry

Eyes of C57BL/6 J or Ano4^KOCfa^ were fixated in Davidson fixative and embedded in paraffin. 5 µm sections were deparaffinized and antigen retrieval was performed by Proteinase K incubation. After blocking the tissue with 5% BSA, the sections were incubated with an antibody against Ano4 (1:200) overnight at 4 °C. Goat anti rabbit Texas Red (Invitrogen) was used as secondary antibody. Nuclei were counterstained using DAPI (3 µM, Invitrogen). Sections were imaged with an Axio Imager M2 fluorescence microscope (Zeiss,) and data was processed by ZEN lite 2012 Software (Zeiss).

### Statistical analysis

All experiments were repeated at least three times. Mean values were given as mean +/− SEM; n refers to the number of experiments. Statistical difference was tested by ANOVA; statistic significant difference was considered at p values smaller than 0.05. Analyzing the effects of ionomycin on the mutant Ano4 currents, we applied the parametric t-test for normally distributed values (according to the Shapiro Wilk Test) and the Wilcoxon Signed Rank Test when the normality test failed.

## Supplementary information

Supplementary Information

## Data Availability

The datasets generated during and/or analyzed during the current study are available in the Zenodo repository (10.5281/zenodo.1484550).
