# Supplementary material for: Anoctamin-4 is a bona fide Ca2+-dependent non-selective cation channel
Source: Sci Rep. 2019 Feb 19;9:2257. doi: 10.1038/s41598-018-37287-y (PMC6381168; doi:10.1038/s41598-018-37287-y)
Supplement: Supplementary file 1 — Supplementary Information [file 41598_2018_37287_MOESM1_ESM.pdf]

**Anoctamin-4 is a bona fide $Ca^{2+}$-dependent non-selective cation channel**

Nadine Reichhart[1, *], Simon Schöberl[2, *], Susanne Keckeis[1], Ahmad S. Alfaar[1], Christophe Roubeix[1], Magdalena Cordes[1], Sergio Crespo-Garcia[1,3], Akvile Haeckel[4], Norbert Kociok[1], Renate Föckler[5], Gabriele Fels[1], Anja Mataruga[6] , Robert Rauh[7], Vladimir M. Milenkovic[8], Kerstin Zühlke[9], Enno Klussmann[9], Eyk Schellenberger[4], Olaf Strauß[1]

[1]Experimental Ophthalmology, Department of Ophthalmology, Charité – Universitätsmedizin Berlin, a corporate member of Freie Universität, Humboldt-University, the Berlin Institute of Health, Berlin, Germany

[2]Department of Gastroenterology, Endocrinology, Rheumatology and Infectious Diseases, University Hospital Regensburg, Regensburg, Germany

[3]Department of Ophthalmology, Maisonneuve-Rosemont Hospital Research Centre, University of Montreal, Quebec, Canada.

[4]Institute for Radiology and Children's Radiology, Charité – Universitätsmedizin Berlin, a corporate member of Freie Universität, Humboldt-University, the Berlin Institute of Health, Berlin, Germany.

[5]Experimental Ophthalmology, Eye Hospital, University Medical Center Regensburg, Regensburg, Germany.

[6]Institute of Complex Systems, Zelluläre Biophysik (ICS-4), Forschungszentrum Jülich, Jülich, Germany

[7]Institut für Zelluläre und Molekulare Physiologie, Friedrich-Alexander-Universität Erlangen-Nürnberg (FAU), Erlangen, Germany.

[8]Department of Psychiatry and Psychotherapy, Molecular Neuroscience, University of Regensburg, Germany
[9] Max Delbrück Center for Molecular Medicine Berlin (MDC), Berlin, Germany

*These authors contributed equally to this study.

**Corresponding Author:**
Olaf Strauß, PhD
Experimental Ophthalmology
Department of Ophthalmology
Charité – Universitätsmedizin Berlin, a corporate member of Freie Universität, Humboldt-University, the Berlin Institute of Health

Campus Virchow Clinic
Augustenburger Platz 1
13353 Berlin
Tel.: +4930450654359
Fax: +4930450554907
olaf.strauss@charite.de

# Supp.Fig.1

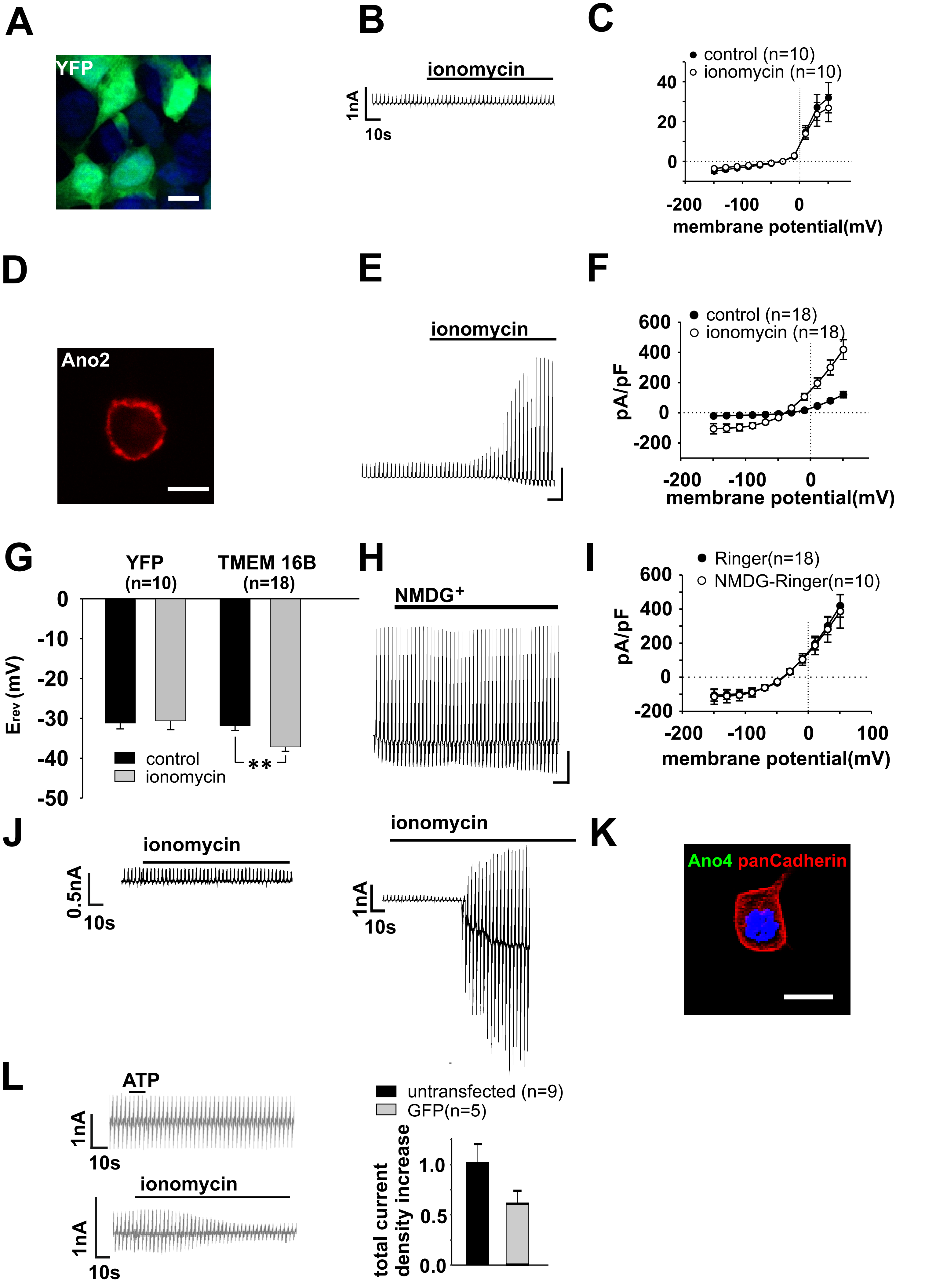
A
YFP
B
ionomycin
1nA
10s
C
control (n=10)
ionomycin (n=10)
40
20
0
-200
-100
0
membrane potential(mV)
D
Ano2
E
ionomycin
F
control (n=18)
ionomycin (n=18)
600
400
200
0
-200
pA/pF
-200
-100
0
membrane potential(mV)
G
YFP
(n=10)
TMEM 16B
(n=18)
0
-10
-20
-30
-40
-50
Erev (mV)
control
ionomycin
**
H
NMDG+
I
Ringer(n=18)
NMDG-Ringer(n=10)
600
400
200
0
-200
pA/pF
-200
-100
0
100
membrane potential(mV)
J
ionomycin
0.5nA
10s
ionomycin
1nA
10s
K
Ano4
panCadherin
L
ATP
1nA
10s
ionomycin
1nA
10s
untransfected (n=9)
GFP(n=5)
total current
density increase
1.0
0.5
0.0

# Supp.Fig.2

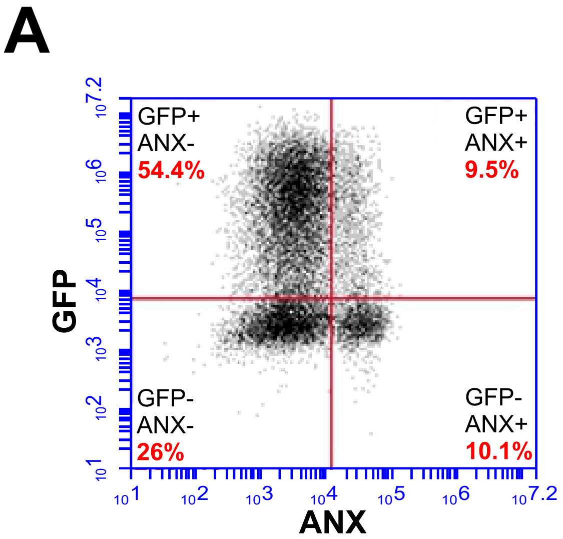
A
GFP
ANX
GFP+
ANX-
54.4%
GFP+
ANX+
9.5%
GFP-
ANX-
26%
GFP-
ANX+
10.1%

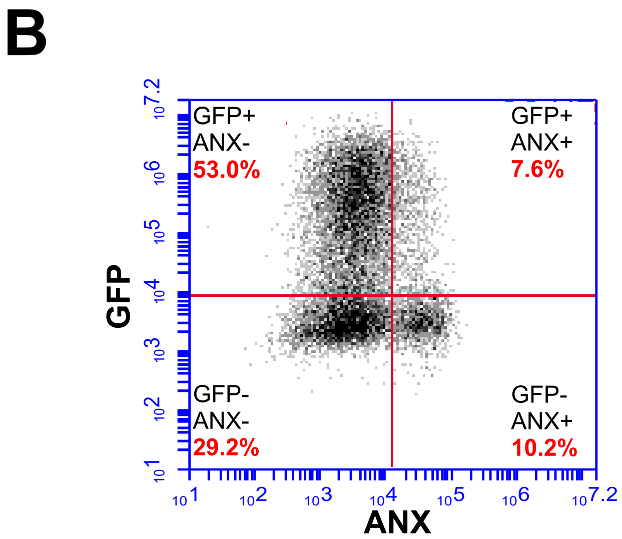
B
GFP
ANX
GFP+
ANX-
53.0%
GFP+
ANX+
7.6%
GFP-
ANX-
29.2%
GFP-
ANX+
10.2%

# Supp.Fig.3

## A

```
hsAno4      768    GIWYGILEGIGILSVITNAFVIAITSDFI    797
rnAno4      768    GIWYGILEGIGILSVITNAFVIAITSDFI    797
mmAno4      768    GIWYGILEGIGILSVITNAFVIAITSDFI    797
btAno4      733    GIWYGILEGIGILSVITNAFVIAITSDFI    762
ecAno4      781    GIWYGILEGIGILSVITNAFVIAITSDFI    810
ggAno4      775    GIWYGILEGIGILSVITNAFVIAVTSDFI    804
laAno4      794    GIWYGILEGIGILSVITNAFVIAITSDFI    823
mamuAno4    768    GIWYGILEGIGILSVITNAFVIAITSDFI    797
```

## B

```
hsAno1    724    GLTPEYMEMIIQFGFVTLFVASFPLAPLFALLNNIIEIRLDAKKFVTELRRP
rnAno1    777    GLTPEYMEMIIQFGFVTLFVASFPLAPLFALLNNIIEIRLDAKKFVTELRRP
mmAno1    754    GLTPEYMEMIIQFGFVTLFVASFPLAPLFALLNNIIEIRLDAKKFVTELRRP
hsAno2    740    GLTPEYMEMIIQFGFVTLFVASFPLAPVFALLNNVIEVRLDAKKFVTELRRP
rnAno2    738    GLTPEYMEMIIQFGFVTLFVASFPLAPVFALLNNVIEVRLDAKKFVTELRRP
mmAno2    738    GLTPEYMEMIIQFGFVTLFVASFPLAPVFALLNNVIEVRLDAKKFVTELRRP
hsAno4    708    GLFDEYLEMILQFGFTTIFVAAFPLAPLLALLNNIIEIRLDAYKFVTQWRRP
rnAno4    708    GLFDEYLEMILQFGFTTIFVAAFPLAPLLALLNNIIEIRLDAYKFVTQWRRP
mmAno4    708    GLFDEYLEMILQFGFTTIFVAAFPLAPLLALLNNIIEIRLDAYKFVTQWRRP

hsAno1    776    VAVRAKDIGIWYNILRGIGKLAVIINAFVISFTSDFIPRLVYLYMYSKNG--
rnAno1    830    VAIRAKDIGIWYNILRGVGKLAVIINAFVISFTSDFIPRLVYLYMYSQNG--
mmAno1    807    VAIRAKDIGIWYNILRGVGKLAVIINAFVISFTSDFIPRLVYLYMYSQNG--
hsAno2    793    DAVRTKDIGIWFDILSGIGKFSVISNAFVIAITSDFIPRLVYQYSYSHNG--
rnAno2    791    DAVRTKDIGIWFDILSGIGKFSVIINAFVIAVTSDFIPRLVYQYSYSHNG--
mmAno2    791    DAVRTKDIGIWFDILSGIGKFSVIINAFVIAVTSDFIPRLVYQYSYSHNG--
hsAno4    760    LASRAKDIGIWYGILEGIGILSVITNAFVIAITSDFIPRLVYAYKYGPCAGQ
rnAno4    760    LASRAKDIGIWYGILEGIGILSVITNAFVIAITSDFIPRLVYAYKYGPCAGQ
mmAno4    760    LASRAKDIGIWYGILEGIGILSVITNAFVIAITSDFIPRLVYAYKYGPCAGQ

hsAno1    828    -------TMHGFVNHTLSSFNVSDFQNGTAPND
rnAno1    882    -------TMHGFVNHTLSSFNVSDFQNGTAPND
mmAno1    859    -------TMHGFVNHTLSSFNVSDFQNGTAPND
hsAno2    845    -------TLHGFVNHTLSFFNVSQLKEGTQPEN
rnAno2    843    -------TLHGFVNHTLSFFNVSQLKEGTQPEN
mmAno2    843    -------TLHGFVNHTLSFFNVSQLKEGTQPEN
hsAno4    812    GEAGQKCMVGYVNASLSVFRISDFENRSEPES
rnAno4    812    GEAGQKCMVGYVNASLSVFRISDFENRSEPES
mmAno4    812    GEAGQKCMVGYVNASLSVFRISDFENRSEPES
```

# Supp.Fig.4

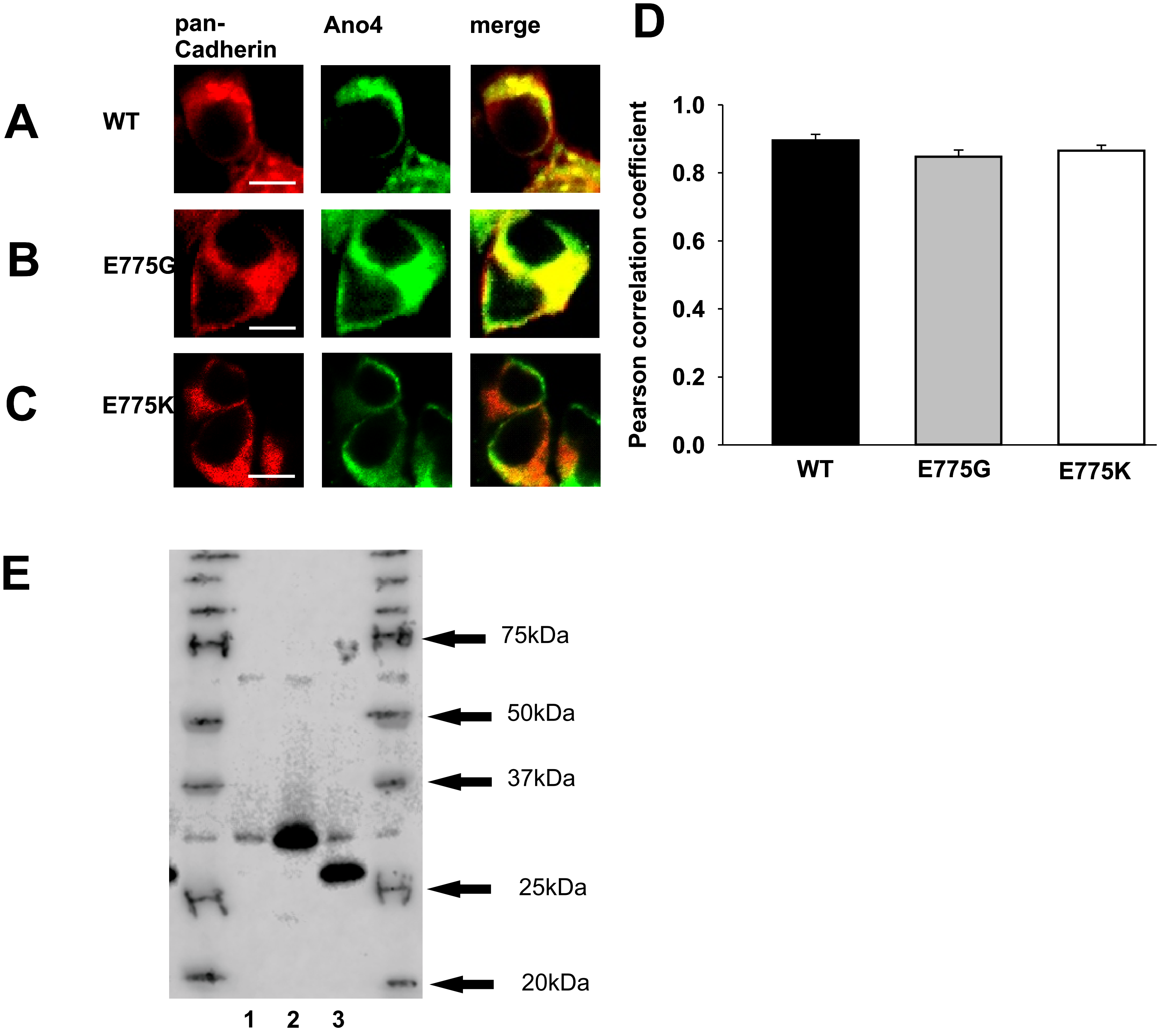
pan-Cadherin
Ano4
merge
A
WT
B
E775G
C
E775K
D
Pearson correlation coefficient
1.0
0.8
0.6
0.4
0.2
0.0
WT
E775G
E775K
E
75kDa
50kDa
37kDa
25kDa
20kDa
1
2
3

**Supp.Fig.5**

**A**

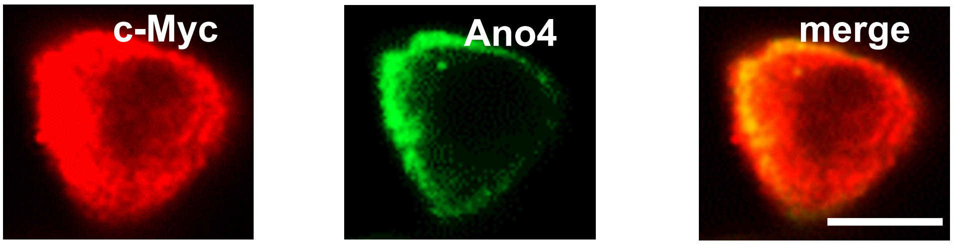

# Supp.Fig.6

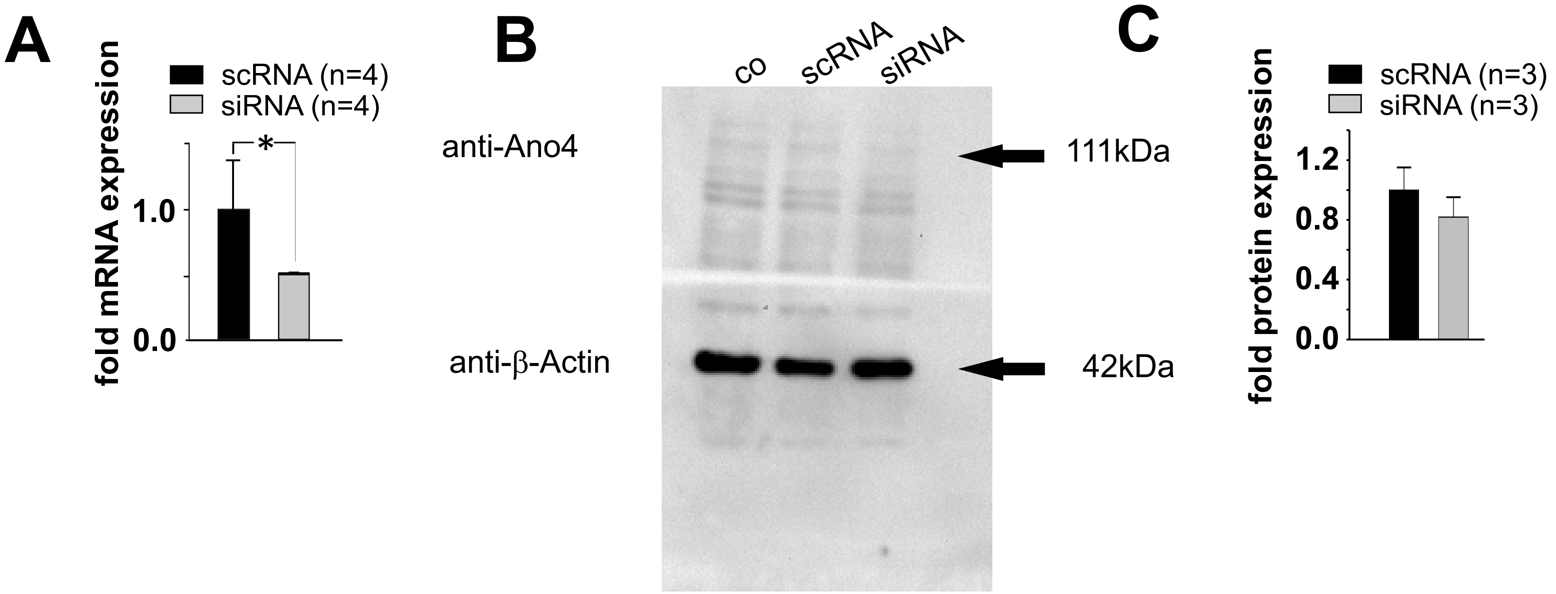
A
fold mRNA expression
scRNA (n=4)
siRNA (n=4)
*
1.0
0.0
B
co
scRNA
siRNA
anti-Ano4
111kDa
anti-β-Actin
42kDa
C
fold protein expression
scRNA (n=3)
siRNA (n=3)
1.2
0.8
0.4
0.0

# Supp.Fig.7

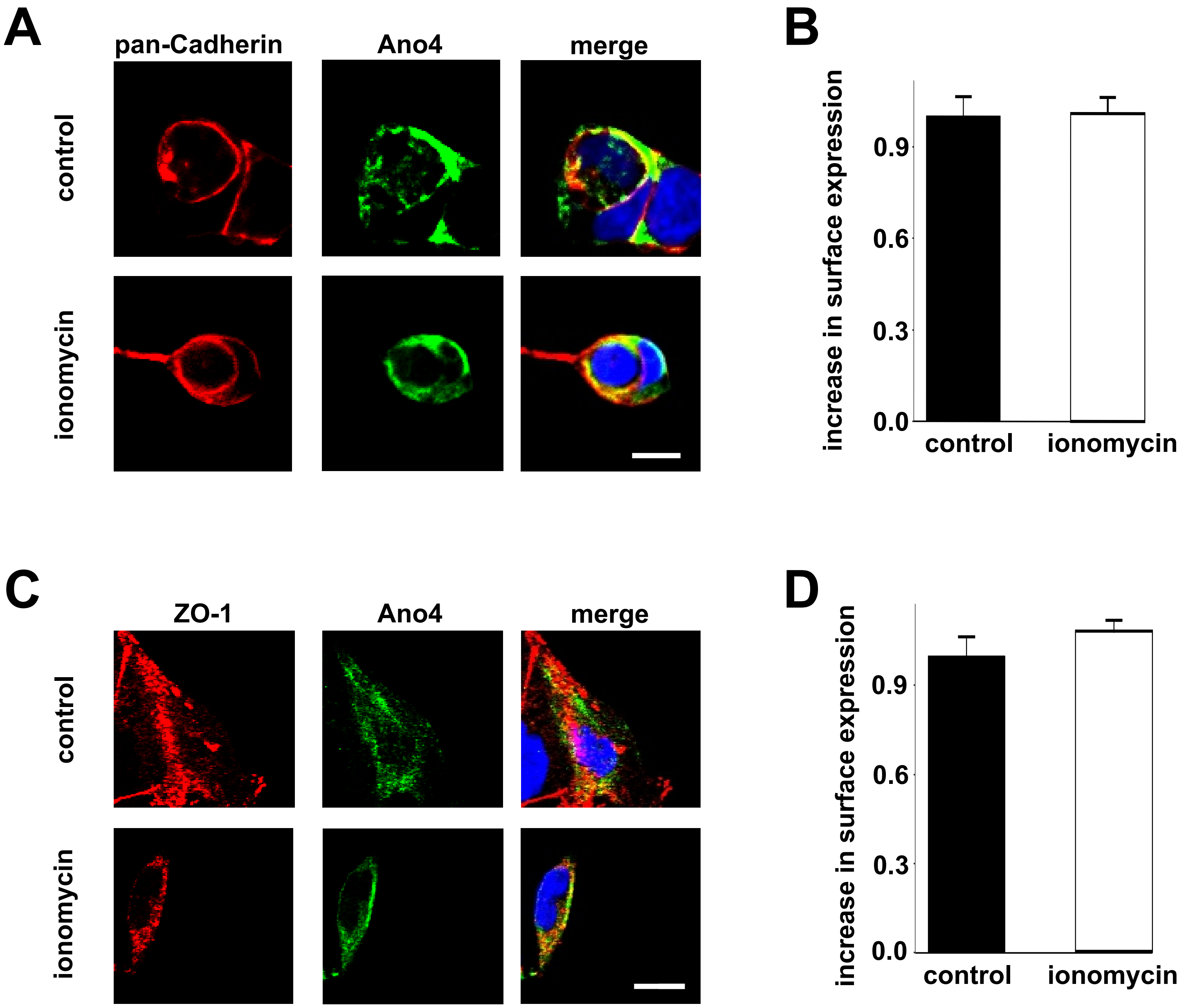
A
pan-Cadherin
Ano4
merge
control
ionomycin
B
increase in surface expression
0.0
0.3
0.6
0.9
control
ionomycin
C
ZO-1
Ano4
merge
control
ionomycin
D
increase in surface expression
0.0
0.3
0.6
0.9
control
ionomycin

**Supplement Table1:**

| Name | Species | Accession number | Length in amino acids |
|---|---|---|---|
| hsAno4 | Homo sapiens | NP_001273544.1 | 955 |
| mmAno4 | Mus musculus | NP_001264117.1 | 955 |
| rnAno4 | Rattus norvegicus | XP_008763453.1 | 955 |
| btAno4 | Bos taurus | NP_001095520.1 | 920 |
| eqAno4 | Equus caballus | XP_005606787.1 | 968 |
| ggAno4 | Gallus gallus | XP_425452.4 | 962 |
| laAno4 | Loxodonta africana | XP_003405674.1 | 981 |
| mamuAno4 | Macaca mulatta | XP_001090523.1 | 955 |
| hsAno2 | Homo sapiens | NP_001265525.1 | 1003 |
| mmAno2 | Mus musculus | NP_705817.2 | 1002 |
| rnAno2 | Rattus norvegicus | XP_008761521.1 | 1002 |
| hsAno1 | Homo sapiens | NP_060513.5 | 986 |
| mmAno1 | Mus musculus | NP_848757.4 | 1017 |
| rnAno1 | Rattus norvegicus | NP_001101034.1 | 1040 |

**Supplement Table1: detailed amino acid sequence information of Ano1, 2 and 4**

name, species, accession number and number of amino acids. hs = homo sapiens; mm= mus musculus; rn = rattus norvegicus; bt = bos taurus; eq = equus caballus; gg = gallus gallus; la = loxodonta africana; mamu = macaca mulatta

**Supplement Figure 1: Electrophysiological analysis of heterologously expressed GFP and Ano2**

**S1A:** Confocal image of HEK cells expressing YFP. Staining with antibody against GFP (green). Scale bar represents 10µm. **S1B:** Raw currents through HEK293 cells heterologously expressing YFP before and after application of ionomycin (1µM), indicated by the bar. **S1C:** Current density-voltage plot of HEK293 cells heterologously expressing YFP before (filled circles) and after application of ionomycin (open circles). Values are given as mean ±SEM. **S1D:** Confocal image of HEK293 cells expressing Ano2-c-Myc. staining with antibody against c-Myc (red). Scale bar represents 10µm. **S1E:** Raw currents through HEK293 cells heterologously expressing Ano2 before and after application of ionomycin (1µM), indicated by the bar. **S1F:** Current density-voltage plot of HEK293 cells heterologously expressing Ano2 before (filled circles) and after application of ionomycin (open circles). **S1G:** Reversal potentials of the data shown in C (left) and F (right) before (black bars) and after (grey bars) application of ionomycin). **S1H:** Raw currents through HEK293 cells heterologously expressing Ano2 before and after application of Ringer-NMDG, indicated by the bar. **S1I:** Current density-voltage plot of HEK293 cells heterologously expressing Ano2 before (filled circles) and after application of ionomycin (open circles). **S1J**: Currents evoked in transfected HEK cells by ionomycin: left panel: cell transfected with Ano4-1-1150del-GFP that lacks the N-terminus of Ano4; right panel cell transfected with Ano4-GFP that contains a GFP at the C-terminus. **S1K:** Confocal image of untransfected HEK293 cells. Staining with antibodies against Ano4 (green) and pan Cadherin(red). Scale bar represents 20µm. **S1L:** *left side:* Currents evoked in untransfected HEK cells (upper panel) by ATP and Hek cells transfected with GFP (lower panel) by ionomycin. *Right side:* Bar chart of total current density increase after the application of ATP in untransfected HEK293 cells (black bar) or ionomycin in HEK293 cells transfected with GFP (grey bar) normalized to current density before ATP stimulation (=1). All values are given as mean ±SEM. **: $p<0.01$.

**Supplement Figure 2: Lack of Scramblase activity of heterologously expressed Ano4 after stimulation with ATP**

**S2A-C:** Assessment of scramblase activity by FACS sorting of annexin A5-labeled HEK293 cells transfected with GFP alone (**A**), with Ano4 plus GFP under control conditions (**B**) or 20 min after the application of ATP (500 µM). (**C**): X-axis: Fluorescence intensity of Anx A5-6S-IDCC (log); Y-axis: Fluorescence intensity of GFP (log). The right upper square represents the transfected, Annexin A5-positive cell fraction.

**Supplement Figure 3: Alignment of conserved domains of Ano1, 2 and 4**

**S3A:** Multiple sequence alignment of Ano4 in the region displayed in Figure 3B. For detailed sequence information see Table1. Highly conserved E (at position 775 in mmAno4) marked in green. **S3B:** Multiple sequence alignment of Anoctamin1, 2 and 4 in the area spanning from amino acid 708-843 (according to Figure 3A). Conserved amino acids are marked in black. Negatively charged amino acids highlighted in green and positively charged amino acids in red.

**Supplement Figure 4: Subcellular localization of WT Ano4, E775G and E775K**

**S4A-C:** Confocal image of HEK cells expressing Ano4 (**A**), E775G (**B**) and E775K (**C**). Staining with antibody against *pan*-Cadherin, a cell surface marker (red, left panel), Ano4 (green, middle panel).right panel: Merge of the two channels. Scale bar represents 10µm. **S4D:** Pearson Correlation Coefficient (PCC) of Ano4 and *pan*-Cadherin in HEK cells transfected with WT Ano4 (black bar), E775G (grey bar) and E775K (white bar). n=11-14 **S4E**: Western Blot of biotinylated samples: lane 1: untransfected HEK293; lane2: HEK293 transfected with GFP; lane 3:HEK293 transfected with GFP; arrows indicate different protein sizes

**Supplement Figure 5: Evaluation of the Ano4 antibody**

**S5A:** Confocal image of a HEK293 cell transfected with Ano4-c-Myc. Staining against c-Myc (red) and Ano4 (green); merge of the channels (yellow). Nuclei were stained with DAPI. Scale bar represents 10µm.

**Supplement Figure 6: Modulation of gene and protein expression of Ano4 by siANO4**

**S6A**: Bar chart comparing the relative expression of Ano4 mRNA in siRNA-transfected cells (grey bar) to scRNA (black bar) transfection. Ano4 expression in scRNA-transfected cells is normalized to 1. **S6B:** Western Blot analysis of lysates of ARPE-19 cells, either non-transfected (co) or transfected with scRNA or siANO4 using an antibody against Ano4. β-Actin served as loading control. (estimated protein size: Ano4 111kDa, β-Actin 42kDa). **S6C:** Densitometric analysis of S5B: Relative expression of Ano4 protein in siAno4-transfected (grey bar) to scRNA-transfected cells (black bar) resulting from Western Blot experiments. Ano4-expression of scRNA transfected cells was normalized to 1. n=3*: $p<0.05$.

**Supplemental Figure 7: Influence of ionomycin on membrane expression of Ano4**

**S7A:** Confocal images of HEK 293 cells heterologously expressing Ano4 after stimulation with control substance (PBS, upper panel) or ionomycin (1µM, lower panel for 5 min. Staining with antibody against *pan*-Cadherin, a cell surface marker (red, left panel), Ano4 (green, middle panel). Right panel: Merge of the channels. Nuclei were stained with DAPI. Scale bar represents 10µm. **S7B:** Bar chart depicting the increase in membrane expression of Ano4 upon ionomycin stimulation. Values are normalized to control. $n_{PBS}$= 29; $n_{ionomycin}$ =26. **S7C:** Confocal images of ARPE 19 cells endogenously expressing Ano4 after stimulation with control substance (PBS, upper panel) or ionomycin (1µM, lower panel for 5 min. Staining with antibody against ZO-1, a cell surface marker (red, left panel), Ano4 (green, middle panel). Right panel: Merge of the channels. Nuclei were stained with DAPI. Scale bar represents 10µm. **S7D:** Bar chart depicting the increase in membrane expression of Ano4 upon ionomycin stimulation. Values are normalized to control. $n_{PBS}$= 12; $n_{ionomycin}$ =20.
